# Prevalence of sickle cell trait and needs assessment for uptake of sickle cell screening among secondary school students in Kampala City, Uganda

**DOI:** 10.1371/journal.pone.0296119

**Published:** 2024-01-19

**Authors:** Shamim Namukasa, Rose Maina, Sarah Nakaziba, Grace Among, Lydia Asasira, Posiano Mayambala, Joseph Atukwatse, Mary Namuguzi, Ahmed M. Sarki

**Affiliations:** 1 School of Nursing and Midwifery, Aga Khan University, Kampala, Uganda; 2 School of Nursing and Midwifery, Aga Khan University, Nairobi, Kenya; 3 Mengo Hospital Blood Bank, Kampala, Uganda; 4 Family and Youth Health Initiative (FAYOHI), Duste, Jigawa State, Nigeria; Faculty of Medicine, University of Belgrade, SERBIA

## Abstract

**Background:**

Sickle cell disease (SCD) is one of the most frequent and traumatizing genetic disease in Uganda, with the prevalence of the sickle cell trait (SCT) estimated at 13.3% leading to serious psycho-social and economic impact on the patients and their families.

**Aim:**

This study aimed to determine the burden of SCT and factors influencing the uptake of screening services among secondary school students in Uganda.

**Methods:**

We used an analytical cross-sectional design with a multi-stage sampling approach. A total of 399 students from four secondary schools in Kampala City were enrolled in this study. Data were gathered using semi-structured questionnaires and blood screening. We used the sickling test to determine the presence of sickle cell alleles among the participants and hemoglobin electrophoresis as a confirmatory test. Data gathered using the questionnaire were analyzed using descriptive and inferential statistics.

**Results:**

In total, 5.8% of participants who were tested during this study had SCT. Most (80.2%) participants were not in an intimate relationship at the time of data collection. The majority (60.4%) had moderate knowledge about SCT screening and obtained information about screening from the school. Only 29 (7.3%) participants knew of a family member with sickle cell. Overall, participants had a negative attitude toward SCT screening (67%), although 41.6% believed that most people who were sickle cell carriers did not live long and were often sick. Statistically significant associations were found between testing for SCT and knowing a partner’s sickle cell status (odds ratio [OR] 2.112, p = 0.043) and Anglican religion (OR 2.075, p = 0.047).

**Conclusion:**

Despite the moderate level of knowledge and negative attitudes, a relatively large number of participants had SCT. This highlights the need for a comprehensive health education package targeting adolescents to promote SCD/SCT screening.

## Background

Sickle cell trait (SCT) is a carrier state arising from the inheritance of one sickle cell “S” allele from one parent and one normal hemoglobin (Hb) “A” allele from the other parent. A person with SCT or heterozygote (HbAS) typically does not show symptoms of sickle cell disease (SCD) or mutated homozygote (HbSS), although they may pass the trait on to their offspring [1]. Globally, SCD is the most prevalent genetic blood disorder and is characterized by progressive organ damage coupled with episodes of acute illness [2, 3]. An estimated 312,000 infants are born with SCD each year, meaning the disease is associated with a significant burden on communities globally [4]. However, only 10% of all patients with SCD are in the developed world [5]. Sub-Saharan Africa contributes more than 75% of all SCD cases, which is expected to increase by 2050 [6]. This projected increase in SCD necessitates more aggressive interventions to help reduce the number of children born with SCD [6, 7].

Although newborn sickle cell screening programs have been implemented in wealthy nations, sub-Saharan Africa lags, with many countries lacking the necessary infrastructure for screening [8]. In addition, in many African communities, SCD causes significant stigmatization of mothers of affected children, and screening can sometimes create discord in homes that may result in marital violence or broken marriages [9]. Low knowledge of SCD, such as people assuming that their spouses were not carriers or that their children could not have SCD, is another factor for the low uptake of SCD screening programs [10, 11]. In addition, some people believed SCT screening was a difficult and painful process, and others thought it would be challenging to persuade their spouses to undergo screening [10]. Other problems included the worry that they would miss out on a "good" spouse if their results were favorable and the worry that their relationship would end if their partner discovered their unusual genotype [12], and the lack of awareness of the occurrence of SCD in their family [10]. Although many teenagers are in relationships that could end in teenage pregnancies, marriage, and children, poor sickle cell screening uptake among young people may mean that adolescents are unaware of their SCT status.

Uganda has the fifth highest sickle cell burden in Africa, with 13.3% of children having SCT and 5,000–20,000 babies born with SCD each year, of which 80% die before reaching 5 years of age [13]. The prevalence of SCT ranges across Uganda, with reported rates of 12.8%–19.8% in Central Uganda and 13.6% in Kampala District [14]. Despite efforts to implement a free screening program for SCD or SCT for newborns and infants under age 2 years in high HIV-burden districts in Uganda [15], social barriers exist for improving SCD care and outcomes, including an overall lack of awareness about SCD, understanding of SCD as a medical condition, and its inheritance. It has been also reported that there is poor parental knowledge of the genetic cause of SCD, awareness of the potential for transmission of SCD to one’s children, and knowledge of one’s own sickle cell status. In addition, families may have to meet the costs associated with screening if they wish to determine their sickle cell status. Moreover, the confirmatory test (Hb electrophoresis) is relatively expensive and limited to a few health facilities. Therefore, most Ugandans only discover they have the sickle cell allele when they have given birth to a child with SCD [16].

A few young people in Uganda know their sickle cell status and knowledge of the disease and preventive measures is limited, many secondary school students may be likely to start relationships with no awareness of their sickle cell status. This increases the likelihood of more children born with SCD, even though a simple blood test can easily detect carriers of the disease. Following screening, couples can receive the proper counseling and information regarding their odds of having affected children. As a result, it’s important to ascertain the prevalence of SCT and assess the variables affecting secondary school students in Kampala City’s uptake of SCT screening. The goal of managing SCD as a priority disease will advance as a result of this information.

## Methods and materials

### Study design

An analytical cross-sectional design was used to determine the prevalence of SCT and factors influencing screening among secondary school students. Quantitative data were collected using semi-structured questionnaires that covered knowledge about and attitudes toward SCT and factors influencing the uptake of SCT screening among secondary school students in Kampala City, Uganda. The prevalence of SCT was determined by testing blood samples that were taken from the students.

### Inclusion & exclusion criteria

The target population included all secondary school students at an advanced level of education studying in day schools in Kampala District, which is in the central region of Uganda. This region has one of the highest burdens of SCT in Uganda at 12.8%–19.8% [14]. The district contains 149 registered secondary schools, of which 133 schools cater to advanced-level students. All students who were present during the study period (2022 academic year). We excluded students who had acute illnesses, had received blood transfusions within the previous 3 months (because the Hb in transfused blood is dominant and may obscure the true picture), had bleeding tendencies, were too weak to participate in this study, or were too busy to devote the necessary time to complete the questionnaire.

### Sample size

The sample size was estimated based on a design effect of 2 and multi-stage sampling. The minimum sample size required was 356 students. To cater for non-response, this minimum sample size was adjusted by 10% (356 × 10% = 35.6), meaning we recruited an additional 36 participants. Therefore, a total sample of 392 participants was considered the minimum sample required for this study.

### Ethics

Ethical approval for this study was sought from the School of Medicine Research and Ethics Committee, Makerere University (Mak-SOMREC-2021-55), and the Uganda National Council for Science and Technology (SS818ES). Permission to conduct the study was sought from the Kampala District Health Office, the District Education Officer, and the administration of each school. An information letter that explained the purpose of this study was sent to the parents of the students offering them the opportunity for them to opt out of having their children participate in testing. In cases when a student was identified with a problem during this study, a referral was made for appropriate clinical management. Written informed consent was obtained from the participants aged 18 years and above, where a participant was <18 years old, we obtained written informed consent from their legal guardians/parents and written assent from the participants. Only participants who provided informed consent were included in the study. Because this study was carried out after COVID and schools had been shut down for two years, some of the participants were older than what was typical for secondary school students.

### Data collection

The participants were given information about the study for them to consent/assent before participation. After providing consent, participants completed a self-administered semi-structured questionnaire covering socio-demographic characteristics, knowledge, attitudes, and factors influencing the uptake of sickle cell screening services. Thereafter, students whose parents/guardians had consented to sickle cell testing were counseled about the procedure. Four milliliters of blood were taken from each student by venipuncture under an aseptic technique following strict blood sample withdrawal and samples were stored at room temperature (22°C–27°C) [17].

### Laboratory procedures

Two sickle cell tests were performed to determine the prevalence of SCT. The sickling test was performed using sodium metabisulphite to determine those that were positive for the sickle cell allele [17] Those who had a positive sickling test underwent a confirmatory test using Hb electrophoresis [18] for positive results via solubility testing. The Hb electrophoresis test was run in duplicate to check for consistency in the results. Furthermore, to ensure the quality of the test results, all experimental procedures were conducted according to the manufacturer’s instructions (Minicap hemoglobin [E] using the minicap flex-piercing) and results were available in 3–7 days. Known HbAS and HbSS blood samples were used as positive controls, and the remaining blood samples were appropriately disposed of. After counseling, the results were shared with the participant and their parents/guardians. Those with positive SCT results were referred for further clinical management.

### Data analysis

Data were entered and analyzed using SPSS version 20. Univariate analysis was conducted to obtain a general description of the study participants. Categorical variables and factors influencing the uptake of SCT screening services were summarized using frequencies and percentages and then displayed in tables. Continuous demographic variables and the proportion of SCT were summarized as means, medians, standard deviations, inter-quartile ranges, and ranges and presented in percentage frequency distribution tables for description purposes. We scored completed surveys using a previously established method [19]. Participants’ knowledge about SCT was categorized as poor (scores below 50%), moderate (scores of 50%–80%), or excellent (scores above 80%). Attitude responses were scored using a 5-point Likert scale (1 = Strongly Disagree to 5 = Strongly Agree). This scale was converted to a 0–100 scale, where Strongly Disagree = 0, Disagree = 25, Neutral = 50, Agree = 75, and Strongly Agree = 100. Negatively worded items were reverse-scored. A positive attitude was defined as a score of ≥75 out of 100 [19]. Knowledge levels and attitudes were summarized using frequencies and percentages.

All predictor variables with a p-value <0.2 in the bivariate analysis were entered into a binary logistic regression model to identify the predictors of screening for SCT versus no screening for SCT. A probability value less than 0.05 was considered statistically significant.

## Results

In total, 399 students from four secondary schools in Kampala City were enrolled in this study. Most (90.2%) participants were aged 17–20 years (mean age 18±1.191 years), and 51.1% were female. The most common religion was Pentecostal (32.1%), and most (80.2%) participants were not in an intimate relationship at the time of data collection. Moreover, most (78.2%) participants thought it was important to know their boy/girlfriend’s sickle cell status. None of the female participants who had boyfriends knew their partners’ sickle cell status. Of all participants, only six (1.5%) reported having been tested for sickle cell before this study. All six participants who had previously been tested reported that they were negative. About 66.7% of those who claimed to have been tested before were tested in a hospital setting. Among those that were tested, 93.3% did not have the sickle cell allele (HbAA). However, about 5.8% of participants had SCT (HbAS) [Table 1].

**Table 1:** Participants’ sociodemographic characteristics (n = 399).

Variable	Frequency	Percentage (%)
**Age, years**		
Mean 18±1.191		
17–20	360	90.2
21–24	39	9.8
**Gender**		
Male	195	48.9
Female	204	51.1
**Religion**		
Catholic	97	24.3
Muslim	46	11.5
Pentecostal	128	32.1
Anglican	108	27.1
Others*	20	5.0
**Currently in an intimate relationship**		
No	320	80.2
Yes	79	19.8
**Important to know your boy/girlfriend’s sickle cell status**		
I don’t know	50	12.5
No	37	9.3
Yes	312	78.2
**Want to be tested for sickle cell allele**		
Yes	328	82.2
No	71	17.8
**Ever tested for sickle cell**		
No	393	98.5
Yes	6	1.5
**Sickle cell results (n = 6)**		
Negative (normal)	6	100
**Place of testing**		
Hospital	4	66.7
Clinic	1	16.7
Others	1	16.7
**SCT prevalence**		
Negative	306	93.3
Positive	22	6.7
**Hemoglobin genotypes**		
AA	306	93.3
AS	19	5.8
SS	3	0.9

*Others = Hindu, Jehovah’s Witness, Pagan, Seventh Day Adventists. SCT, sickle cell trait.

Most (89.7%) participants who agreed to be tested for sickle cells in this study wanted to know their sickle cell status. Of those who did not want to be tested for sickle cell, about 2.3% indicated this was because of what they perceived sickle cell to be [Table 2].

**Table 2:** Reasons for screening/not screening for sickle cell.

Variable	Frequency	Percentage (%)
**Reasons for accepting to be tested for sickle cell** *		
Know my sickle cell status	358	89.7
Family history SCD	5	1.3
Free of charge	1	0.3
Assist in reproductive health decision-making	32	8.0
**Reasons for declining to be tested for sickle cell** *		
Fear of results	7	1.8
Perceived knowledge about sickle cell	9	2.3
Tested before	4	1.0
No family member has signs	2	0.5
Think it is painful	4	1.0

*Multiple responses permitted. SCD, sickle cell disease.

Perceived knowledge about sickle cell testing: not ready to test, don’t want to give blood, God heals SCD.

Generally, majority (67%) of the participants had a negative attitude toward testing for SCT [Table 3]. Most (83.2%) participants agreed that it was important for both partners (boyfriends and girlfriends) to be tested to confirm their sickle cell status before sexual intercourse/marriage. About one-third (32%) of participants disagreed that two people who have the sickle cell allele (sicklers)/trait should be discouraged from having a child together or marrying each other. Majority (85.7%) of the participants agreed that it was necessary to provide genetic counseling about sickle cell to all people intending to have a serious intimate relationship. In addition, 75.9% of participants agreed that testing all newborn babies for the sickle cell allele was necessary [Table 3].

**Table 3:** Attitudes toward sickle cell screening.

Variable	Strongly Disagree	Disagree	Uncertain	Agree	Strongly Agree
	n (%)	n (%)	n (%)	n (%)	n (%)
**Sickle cell disease can be prevented by**					
Testing of both boyfriend and girlfriend before sexual intercourse/marriage	18 (4.5)	19 (4.8)	30 (7.5)	113 (28.3)	219 (54.9)
Discouraging two people who have the sickle cell allele from having a child together or marrying each other	64 (16.0)	64 (16.0)	56 (14.0)	85 (21.3)	130 (32.6)
Providing genetic counseling about sickle cell to all people intending to have a serious intimate relationship	20 (5.0)	14 (3.5)	23 (5.8)	141 (35.3)	201 (50.4)
Testing all newborn babies for the sickle cell allele	14 (3.5)	33 (8.3)	49 (12.3)	121 (30.3)	182 (45.6)
**Benefit of testing for sickle cell trait**					
It is useful to know if I am a sickle cell carrier	7 (1.8)	5 (1.3)	10 (2.5)	123 (30.8)	254 (63.7)
It is useful to know if my boy/girlfriend is a sickle cell carrier	4 (1.0)	6 (1.5)	13 (3.3)	161 (40.4)	215 (53.9)
I would encourage my boy/girlfriend to be tested for sickle cell if I was found to be a sickle cell carrier	6 (1.5)	14 (3.5)	30 (7.5)	151 (37.8)	198 (49.6)
Knowing the risk of having a child with sickle cell disease would change my pregnancy/marriage plans	39 (9.8)	41 (10.3)	58 (14.5)	108 (27.1)	153 (38.3)
Testing for sickle cell is painful	56 (14.0)	100 (25.1)	85 (21.3)	111 (27.8)	47 (11.8)
I fear getting positive results	59 (14.8)	77 (19.3)	51 (12.8)	131 (32.8)	81 (20.3)
It will be hard to convince my boy/girlfriend to have the test	50 (12.5)	84 (21.1)	64 (16.0)	109 (27.3)	92 (23.1)
Being a sickle cell carrier would make me less confident about forming relationships	54 (13.5)	82 (20.6)	57 (14.3)	108 (27.1)	98 (24.6)
I fear being stigmatized after the results are out	76 (19.0)	70 (17.5)	41 (10.3)	120 (30.1)	92 (23.1)
I would break up with my boy/girlfriend if I found out that she/he is a sickle cell carrier even if I am not	81 (20.3)	78 (19.5)	66 (16.5)	92 (23.1)	82 (20.6)
**Should screening for sickle cell be made widespread**					
I support sickle cell disease testing	19 (4.8)	16 (4.0)	11 (2.8)	79 (19.8)	274 (68.7)
I would want to know my sickle cell status	27 (6.8)	15 (3.8)	18 (4.5)	102 (25.6)	237 (59.4)
Sickle cell testing is important at my present age	61 (15.3)	28 (7.0)	39 (9.8)	81 (20.3)	190 (47.6)
I support sickle cell disease/carrier testing for all people	10 (2.5)	13 (3.3)	10 (2.5)	78 (19.5)	288 (72.2)

**Fig 1:**
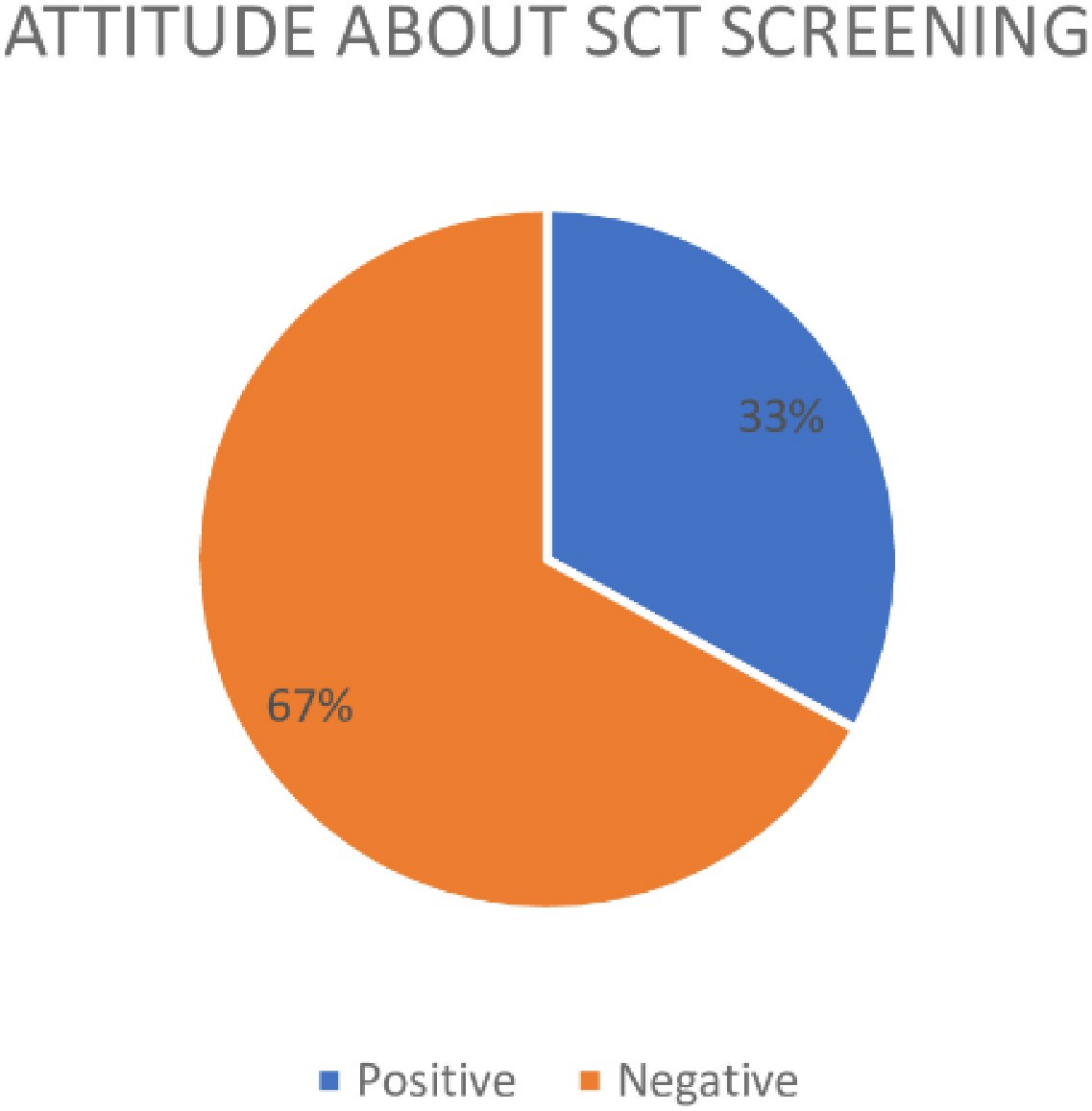
Attitude of secondary school students about SCT screening.

Fig 1 showed that 268 (67%) participants had a negative attitude toward testing for SCT.

A large number (93.9%) of the participants agreed that it would be useful to know if their boy/girlfriend were sickle cell carriers. Majority (87.4%) of the participants agreed they would encourage their boy/girlfriend to be tested for sickle cell if they were found to have SCT. Surprisingly one in five (20.1%) participants disagreed that knowing the risk of having a child with SCD would change their plan to have a child or marry [Table 3].

More than a third (39.6%) of participants reported experiencing discomfort during their sickle cell test, and more than half (53.1%) of the participants expressed fear of positive sickle cell results. It would be difficult to persuade their boyfriend/girlfriend to take the test, according to 50% of participants (50.4%). Additionally, 108 participants (51.7%) felt that having sickle cell anemia would make them less confident in dating, and almost six out of ten (53.4%) participants were concerned about being stigmatized after the results were made public. In addition, despite not having sickle cell disease, 53.7% of participants said they would discontinue their relationship with their boyfriend or girlfriend if they learned they had the disease. Most participants (67.9%) believed that SCT screening was necessary at their current age. Most participants supported SCT testing (88.5%) and indicated that they would like to know their sickle cell status (85%), respectively. Most participants, 72.2%, agreed that SCT/carrier testing ought to be done on everyone [Table 3].

Majority (n = 241, 60.4%) of participants had moderate knowledge about SCD and testing. Only 29 (7.3%) participants knew of a family member with sickle cell. Most (n = 330, 82.7%) participants knew that a carrier/sickler could pass on sickle cell to their children, and the majority (n = 301, 75.4%) stated that the sickle cell allele can be confirmed by a blood test. Around half of the participants (n = 199, 49.9%) obtained information about sickle cell from schools [Table 4].

**Table 4:** Knowledge about sickle cell screening.

Variable	n (%)
**Overall knowledge**	
Poor	58 (14.5)
Moderate	241 (60.4)
Excellent	100 (25.1)
**How to confirm that someone has the sickle cell allele**	
They look sick	23 (5.8)
Urine test	4 (1.0)
By a blood test	301 (75.4)
Don’t know	67 (16.8)
Others	4 (1.0)
**Anyone in your family have sickle cell**	
Don’t know	204 (51.1)
No	166 (41.6)
Yes	29 (7.3)
**A carrier/sickler can pass on sickle cell to their children**	
Don’t know	58 (14.5)
No	11 (2.8)
Yes	330 (82.7)
**Positive response (yes) about the source of information about sickle cell and testing** **	
Radio	39 (9.8)
Television	73 (18.3)
Health camps	34 (8.5)
Posters/fliers	1 (0.3)
Magazines/newspapers	18 (4.5)
School	199 (49.9)
Family	29 (7.3)
Hospital	11 (2.8).
Others*	8 (3.5)

* Others: friends, Internet, online, parents.

** Multiple responses allowed.

A large number (n = 326, 81.7%) of participants stated that people who were sickle cell carriers had inherited it from at least one of their parents but only 150 (37.6%) participants knew that people who were sicklers had inherited it from both parents. In addition, 166 (41.6%) participants stated that most people who were sickle cell carriers did not live long and were often sick, and 180 (45.1%) participants stated that sickle cell carriers had many medical problems. More than half (n = 217, 54.4%) of the participants disagreed that all children of a sickle cell carrier would also be carriers. In addition, 176 (44.1%) participants stated that SCD was not curable. The majority (n = 240, 60.2%) stated that when one parent was a carrier, there was a chance that they would have a child with SCD, and almost three quarter 288 (72.2%) participants said that when both parents were carriers, there was a chance that they would have a child with SCD [Table 5].

**Table 5:** Knowledge about sickle cell disease.

Variable	Don’t know	False	True
	n (%)	n (%)	n (%)
Sickle cell disease is curable	152 (38.1)	176 (44.1)	71 (17.8)
People who are sickle cell carriers inherited it from at least one of their parents	51 (12.8)	22 (5.5)	326 (81.7)
People who are sicklers inherited it from both parents	122 (30.6)	127 (31.8)	150 (37.6)
Most people who are sickle cell carriers live long and healthy lives	110 (27.6)	166 (41.6)	123 (30.8)
Sickle cell carriers have many medical problems	123 (30.8)	96 (24.1)	180 (45.1)
If a person is a sickle cell carrier, all their children will be sickle cell carriers	121 (30.3)	217 (54.4)	61 (15.3)
When one of the parents is a carrier, there is a chance that they will give birth to a child who has sickle cell disease	60 (15.0)	99 (24.8)	240 (60.2)
When both parents are carriers, there is a chance that they will give birth to a child who has sickle cell disease	82 (20.6)	29 (7.3)	288 (72.2)

Statistically significant associations were found between SCT testing and knowing a partner’s sickle cell status (odds ratio [OR] 2.112, p = 0.043) and being of Anglican religion (OR 2.075, p = 0.047) [Table 6]. Participants who thought that it was important to know their partner’s status were two times more likely to screen for SCT than those who did not know that it was important. In addition, participants who were Anglican were two times more likely to screen for SCT compared with participants who were of the Catholic religion. There were no statistically significant association between testing for SCT and whether the participant had been tested before and gender. However, prior testing status (tested before) confounded the relationship between testing and the importance of knowing a partner’s sickle cell status [Table 6].

**Table 6:** Factors associated with sickle cell screening uptake.

Variable	Screening status		Unadjusted OR	p-value	Adjusted OR	95%CI	p-value
	Screened	Not Screened					
	n (%)	n (%)					
**Gender**							
Male	40 (56.3)	155 (47.3)	Ref		1.000	Ref	
Female	31 (43.7)	173 (52.7)	1.44	0.166	1.594	0.929, 2.733	0.090
**Religion**							
Catholic	25 (35.2)	72 (22.0)	Ref		1.000	Ref	
Muslim	6 (8.5)	40 (12.2)	2.563	0.090	2.500	0.920, 6.798	0.073
Pentecostal	22 (31.0)	106 (32.3)	1.792	0.119	1.703	0.879, 3.299	0.114
Anglican	16 (22.5)	92 (28.0)	2.168	0.053	2.075	1.010, 4.260	**0.047** *
Others	2 (2.8)	18 (5.5)	3.841	0.144	3.629	0.759, 17.350	0.106
**Important to know partner’s sickle cell status**							
I don’t know	0 (0.0)	1 (1.4)	Ref		1.000	Ref	
No	20 (100.0)	59 (85.5)	0.776	0.860	0.786	0.296, 2.088	0.629
Yes	0 (0.0)	9 (13.0)	2.191	0.022	2.112	1.023, 4.357	**0.043** *
**Tested before**							
No	323 (98.5)	70 (98.6)	1.000		1.000	Ref	
Yes	5 (1.5)	1 (1.4)	1.084	0.942	0.964	0.106, 8.729	0.974
**Attitude**							
Negative	7 (9.9)	18 (5.5)	1.000		1.000	Ref	
Positive	64 (90.1)	310 (94.5)	1.884	0.174	1.418	0.535, 3.755	0.483

*Statistically significant; OR- odds ratio; CI, confidence interval.

## Discussion

SCD is a debilitating chronic genetic blood disorder that places psychosocial, financial, and frequently painful burdens on affected individuals and their families. Those who had previously been tested said they had received a negative result. Among those examined for this study, 93.3% were normal, 0.9% had HbSS genotype (sicklers), and 5.8% had the HbAS genotype (carriers). This calls for public health interventions that encourage people to undergo screening to confirm their sickle cell status to make informed decisions [20]. A prior study discovered a high prevalence of sickle cell trait, which contrasts with our findings of HbAS (18.7%, 42/225) among secondary school students in Kenya [20]. Another cross-sectional study conducted among secondary school students aged 9–25 years showed that 59% knew their genotype and 11.1% claimed they were AS genotype [21].

Only 19.8% of participants in the present study were currently in an intimate relationship. None of these participants knew their partner’s sickle cell status, although 78.2% of all participants knew that it was important for an individual to know their partner’s status. Because many participants were under 20, there’s a chance they didn’t feel the need to be aware of such information because they don’t often take intimate relationships seriously. This calls for health education programs about SCD targeted at young people. The price of SCD testing could also be a deterrent, as a study from Uganda revealed that testing for SCD was made more difficult by the availability and affordability of testing services [22]. That study also highlighted the need for targeted SCD education to groups such as adolescents who may not think of the possibility of having the sickle cell allele. In the same study, 27.3% of participants reported it would be hard to convince their partner to have the test, although those who knew their partner’s sickle cell status were more likely to undergo testing themselves than those who did not. This could be a reminder of the likelihood of having a child with SCD. Similarly, a study conducted in a division of Kampala, Uganda, showed that most (90.2%) respondents did not know their partner’s genotype [13].

Only 1.5% of participants in our study reported having been previously tested for sickle cells. This might be due to the absence of SCT testing from secondary school or the fact that screening for diseases is not something that people often consider doing, especially if they are not feeling ill. It could also be explained by the fact that SCD testing is only offered in a few specific medical facilities and is not a free service. In this study, most secondary school students did not know their Hb genotype. Participants who had already undergone screening were most frequently tested to determine whether they had sickle cell disease. Among participants who did not want to be tested, six participants (23.1%) feared positive results, feared the pain associated with testing, and indicated that no family member had signs of SCD. Similarly, a cross-sectional study among adults (mean age 29 years) in Muscat, Oman, showed that 36% thought SCT screening was a difficult and painful process, which explained the small percentage who had been screened (24.4%), and 37.8% felt it would be hard to convince their partners to go for screening [23]. In another study, most (94.6%) youths (aged 22–29 years) in South Nigeria knew their Hb genotype (SCD carrier status), with the most common reason for checking their sickle cell status being a school entry requirement [24].

To prevent SCD, one must first be aware of their genotype. In this study, many participants (60.4%) had a basic understanding of SCD and testing. Similarly, a study conducted among youths aged 22–29 years in Nigeria showed that most respondents (63.5%) had a fair knowledge of SCD [24]. This implies that those who have SCD but do not know their genotype may not be able to access care for the disease, thereby increasing SCD-related morbidity and mortality. Most participants in this study had heard about SCD at school. This is presumably because students had easy access to tools like the Internet while they were in school and were also taught about hereditary illnesses. This finding was consistent with previous studies [25, 26] that reported that most participants learned about SCD from media and school. This suggests that schools and the media can be effective institutions and platforms for educating people about SCD. Carriers of the disease who do not know their genotype may not seek the necessary genetic counseling to make informed marital choices. Therefore, school authorities should encourage parents to find out their children’s Hb genotype as well as students older than 18 years [27].

More than eight in ten respondents (82.7%) were aware that carriers can pass sickle cell to their offspring, but more than half (51.1%) were unaware of whether anyone in their family had the condition. This may be because some parents may not reveal certain medical information to their children, especially if there is no one suffering major complications. In addition, a previous study by Okwi, Byarugaba [16] reported that most Ugandans only discovered they had the sickle cell allele when they had a child with SCD yet sickle cell is known to play a causal role in several morbidities, which makes the disclosure process critical [28]. Similar findings were reported in another study Tusuubira, Nakayinga [13] in which more than half (54.0%) of the participants knew SCD was inherited from both parents, and the majority (62.9%) had a family member with SCD. Most participants in the present study knew that there was a chance of having a child with SCD if one parent was a carrier (81.7%) or if both parents were carriers (37.6%). This suggested that many participants had incomplete knowledge regarding the spread of SCD, which could result in unwise health choices. These results concurred with those of an earlier study that found the majority of participants accurately thought that SCD was inherited from parents [21].

Three-quarters of the participants in this study stated that the sickle cell allele can be confirmed by a blood test, and more than half believed SCD was curable. Similarly, in another study, Ghimire [29] found that 63.1% of respondents knew that blood tests could detect the disease and 71.7% mentioned that SCD had treatment. Surprisingly, 30.8% of participants in our survey believed sickle cell carriers did not live long and healthy lives, and more than half (45.1%) believed sickle cell carriers had several medical issues. This demands ongoing teaching about SCD in schools and the public [13], so that those with SCT become knowledgeable of their carrier status, are educated on how they can potentially pass the trait or disease on to their offspring, and better understand the outcomes of SCD.

We discovered that compared to Catholics, Anglicans were twice as likely to have a sickle cell test. This is likely because Anglicanism is one of Uganda’s most prevalent religions, and its priests urge people to have children and use reproductive health care facilities, which go hand in hand with screening for certain diseases like sickle cell disease. Scholars have shown that religious leaders play a vital role in the implementation and development of health interventions that promote the health of people [30, 31]. Some religious organizations educate the public through counseling sessions or seminars, and the majority require genetic counseling before marriage. In other studies, religious bodies were mentioned among the sources of health information [27]. This calls for strengthening the integration of health and religious institutions in the dissemination of health information. Religious organizations have been successfully used in disseminating health information in other disease situations such as HIV/AIDS [27]. As a result, religious groups and healthcare professionals must collaborate on SCD preventive initiatives. If few youths know their sickle cell status and most only have moderate knowledge of the disease, many young people may enter relationships without learning their sickle cell status, which will increase the chances of having children with SCD.

Most (66%) of the participants in this study had a negative attitude toward SCD/SCT and testing. This can be due to Uganda’s general awareness of SCD and continuous public awareness programs. This may lead to stigmatization, have an impact on how sickle cell patients respond to cues, and how they seek care, and ultimately have an impact on patient outcomes. Similar findings have been reported in a Nigerian study that explored the stigmatising attitude of secondary school students towards peers with SCD. It was indicated that a significant proportion of students still had negative attitudes towards peers with SCD despite having had contact with people who have SCD [32]. A sixth (16%) of participants disagreed that two people who have the sickle cell allele (sicklers)/trait should be discouraged from having a child together or marrying each other. In addition, 10.3% disagreed that knowing the risk of having a child with SCD would change their pregnancy/marriage plans.

This might be the case because participants don’t understand the pain and psychosocial trauma that affected children go through and think they are immune to the illness. The beliefs of people about several factors, such as perceived susceptibility to the disease, the severity of the disease, the advantages of screening, and the barriers to screening, have an impact on the uptake of screening services for numerous diseases [33]. Moreover, participants in this study agreed that being a sickle cell carrier would make them less confident about forming relationships (27.1%) and many feared being stigmatized based on their results (30.1%). Communities still have misconceptions about SCD, its cause, management, and outcomes that affect how they view people with SCD. This was supported by similar findings in a study that highlighted stigmatization as a barrier to screening for SCD [34]. In contrast to the above findings, another study reported that more than half (56%) of students agreed they should not marry someone with SCT/SCD irrespective of their genotype [12].

Half (50.4%) of the participants in this study strongly agreed that genetic counseling about sickle cell should be provided to all people intending to have a seriously intimate relationship and if they were found to have SCT, almost half (49.6%) of people would highly advise their boyfriend/girlfriend to get tested for sickle cell. This may be because SCD is a fatal disease and the union between two SCT carriers gives a 25% risk of having a sick child in each pregnancy Maboulou, Ngoutane [35].

Most individuals (72.2%) supported testing for SCD carriers in all people, and the majority (45.6%) strongly agreed that newborns should all be tested for the SCD allele. A free screening program for SCD/SCT for newborns and infants under 2 years in Uganda was established in high HIV-burden districts [15], although mechanisms for informing families were locally determined and may not be systematic. In addition, students/families may have to meet associated screening costs if they are interested in finding out their SCD status. A previous study showed that 55% of youths agreed with legislation prohibiting marriage between trait carriers to prevent the births of babies with SCD [24]. The World Health Organization also advocates for carrier detection and genetic counseling [36], which were reported to be effective in the control of thalassemia in countries such as Cyprus and Iran [37]. Primary preventive measures such as avoidance of carrier marriages/pregnancies through health education, population screening, carrier detection, genetic counseling, and pre-conceptual diagnosis should therefore receive greater emphasis in efforts to control SCD in Uganda.

## Conclusion

Most individuals in this study had negative attitudes toward testing, moderate understanding of SCD/SCT, and moderate knowledge of their genotype. The knowledge of the partner’s status and religion was substantially linked to SCT testing. The participants’ understanding of SCD’s causes and prevention was lacking. Therefore, cooperation between the health and education sectors is necessary for SCD prevention, diagnosis, and management. Regular health education campaigns on SCD are also necessary, as are free Hb electrophoresis tests for teenagers who are enrolled in school. To inform parents about the benefits of early SCD diagnosis for their children through Hb genotype testing, policymakers in the education sector should also focus on Parents Teachers Associations in schools. The nurses at the schools should also periodically teach the students about inherited illnesses like SCD/SCT. The cross-sectional nature of the study design, which prevents the researchers from deducing causality, is one of the study’s shortcomings. The sample was taken from the Central region of Uganda, one of the country’s five geographical regions, which is a conceivably plausible limitation. However, the data from this study will serve as a starting point for additional research. There is a need for more research projects using nationally representative samples from all five geographical regions.

## Supporting information

S1 File Questionnaire. (PDF)

S2 File Summary statistics. (ZIP)

S3 File Laboratory protocol. https://www.protocols.io/file-manager/1219B065DBA911EDBE2A0A58A9FEAC02.
(RAR)

## Abbreviations

Hb: hemoglobin
SCD: sickle cell disease
SCT: sickle cell trait
