# Supplementary material for: Prevalence of sickle cell trait and needs assessment for uptake of sickle cell screening among secondary school students in Kampala City, Uganda
Source: PLoS One. 2024 Jan 19;19(1):e0296119. doi: 10.1371/journal.pone.0296119 (PMC10798531; doi:10.1371/journal.pone.0296119)
Supplement: S1 File — (PDF) [file pone.0296119.s001.pdf]

APPENDIX B: QUESTIONNAIRE

Thank you for accepting to participate in this study titled "**Prevalence of Sickle Cell Trait and Needs Assessment for Up-Take of Sickle Cell Screening among Secondary School Students in Kampala City**". The study consists of four sections. Kindly fill in all the sections appropriately as given by the instructions. Questions will be answered by either ticking the answer you choose or writing in the provided space.

School Name: _______________ ________________________

Code no.________________

SECTION 1: SOCIO-DEMOGRAPHIC DATA

Please **Tick** the answer which best applies to you and fill in suitably where applicable.

1. What is your Gender?
   a) Male

   b) Female

2. How old are you (**in Years)**? ________

3. What is your religion?

a) Catholic
b) Muslim
c) Pentecostal
d) Anglican
e) Others- specify_______________

SECTION 2: UPTAKE OF SICKLE CELL TRAIT TESTING

Please Tick The Answer Which Best Applies To You and Fill In Suitably Where Applicable.

1. Have you ever tested for sickle cell?

a) Yes

b) No

2. Would you wish to be tested for sickle cell?

a) Yes
b) No

3. If **Yes in question 2**, give reasons for your response

____

____

4. If **No in question 2**, give reasons for your response

____

____

5. If yes, where was the test done from?

a) Hospital
b) Clinic
c) Health camp
d) Don't know
e) Others (specify)

6. **If yes in question 5 above**, what were the results?

a) Negative (Normal)
b) Sickle Cell Carrier
c) Sickle Cell Disease
d) I don't know

7. Are you in any intimate relationship currently? **If no**, **go to question 9**

a) Yes
b) No

8. Do you know your boy/girlfriend's sickle cell status?

a) Yes
b) No

9. Do you think it would be important to know your boy/girlfriend's sickle cell status?

a) Yes
b) No
c) I don't know

SECTION 3: KNOWLEDGE ABOUT SICKLE CELL TESTING.

1. Does anyone in your family have **sickle cell (carrier/sickler)**? Q1

e) Yes
f) No
g) I don’t know

2. If a person has **sickle cell (carrier/sickler)**, can it be passed on to his or her children? Q2

a) True
b) False
c) I don’t know

3. People who are **sickle cell carriers** have inherited it from at least one of the parents Q3

a) True
b) False
c) I don’t know

4. People who are **sicklers**, have inherited it from both parents Q4

a) True
b) False
c) I don’t know

5. Most people who are **sickle cell carriers** live long and healthy lives Q5

a) True
b) False
c) I don’t know

6. If a person is a **sickle cell carrier**, all his or her children will be **sickle cell carriers Q6**

a) True
b) False
c) I don’t know

7. **Sickle cell carriers** have many medical problems Q7

a) True
b) False
c) I don’t know

8. Is sickle cell disease curable? Q8

h) Yes
i) No
j) I don’t know

9. How can you confirm that someone has the sickle cell gene? Q9

a) They look sick
b) Urine test
c) By a blood test
d) Don’t know
e) Others-specify________________

10. When one of the parents is carrier, there is a chance that they will give birth a child who has sickler (sickle cell disease)? Q10

a) True 2
b) False
c) I don’t know

11. When both parents are carriers, there is a chance that they will give birth a child who has sickler (sickle cell disease)? Q11

d) True 2
e) False
f) I don’t know

12. Based on the information you have provided above, what were your sources of information about sickle cell? Q12

a) Radio
b) Television
c) Health camps
d) Posters/fliers
e) Magazines/newspapers
f) School
g) Family
h) Hospital
i) Others Specify _________________

SECTION 4: ATTITUDES TOWARDS SICKLE CELL TRAIT TESTING

Please read each question very carefully and then tick only one of the following options; strongly disagree, disagree, uncertain, agree, and strongly agree.

		**Question**	**Strongly Disagree**	**Disagree**	**Uncertain**	**Agree**	**Strongly Agree**
		**Sickle cell disease can be prevented by**					
1	Q13	Testing of both boy and girl friend before sexual intercourse/marriage					
2	Q14	Discouraging two people who have the sickle cell gene from having a child together or marrying each other					
3	Q15	Providing genetic counseling about sickle cell to all people intending to have a serious intimate relationship					
4	Q16	Testing all newly born babies for the sickle cell gene					
		**Benefit of testing for Sickle cell trait**					
5	Q17	It is useful to know if I am a sickle cell carrier					
6	Q18	It is useful to know if my boy/girl friend is a sickle cell carrier					
8	Q19	I would encourage my boy/girl friend to be tested for the sickle cell if I was found to be a sickle cell carrier					
9	Q20	Knowing the risk of having a child with sickle cell disease would change my pregnancy/marriage plans					

		**Barriers to testing for sickle cell**
10	Q21	Testing for the sickle cell is painful
11	Q22	I fear getting positive results
12	Q23	It will be hard to convince my boy/girl friend to have the test

13	Q24	Being a sickle cell carrier would make me less confident about forming relationships
14	Q25	I fear being stigmatized after the results are out
15	Q26	I would break with my boy/girl friend is I found out that she/he is a sickle cell carrier even if I am
		**Should screening for sickle cell be made widespread**
16	Q27	I support sickle cell disease testing
17	Q28	I would want to know my sickle cell status
18	Q29	I don't think sickle cell testing is important at my present age
19	Q30	I support sickle cell disease carrier testing for all people
